# Aortic valve surgery for aortic regurgitation caused by Libman-Sacks endocarditis in a patient with primary antiphospholipid syndrome: a case report

**DOI:** 10.1186/s13019-021-01458-2

**Published:** 2021-04-17

**Authors:** Yan Le Ho, Nurul Atiqah Ahmad Zaidi, Ahmadi Salleh, Basheer Ahamed Abdul Kareem

**Affiliations:** 1 Department of Cardiothoracic Surgery, Penang General Hospital, Jalan Residensi, 10990 George Town, Pulau Pinang Malaysia; 2 Department of Pathology, Penang General Hospital, Jalan Residensi, 10990 George Town, Pulau Pinang Malaysia

**Keywords:** Antiphospholipid syndrome, Antiphospholipid antibodies, Libman-sacks endocarditis, Cardiac manifestations, Heart valve disease, Aortic regurgitation, Valve replacement surgery

## Abstract

**Background:**

Antiphospholipid syndrome is an antibody mediated pro-thrombotic state leading to various arterial and venous thromboses. The syndrome can be either primary or secondary to other autoimmune diseases, commonly systemic lupus erythematosus. Cardiac involvement, in particular valvular disease is common in patients with antiphospholipid syndrome, occurring in about a third of these patients. Valvular diseases associated with antiphospholipid syndrome often occur as valve thickening and non-bacterial vegetation or Libman-Sacks endocarditis. Deposits of antiphospholipid immunoglobulin and complement components are commonly observed in the affected valves, suggesting an inflammatory process resulting in valvular vegetation and thickening. Libman-Sacks endocarditis has a high propensity towards mitral valve, although haemodynamically significant valvular dysfunction is rare.

**Case presentation:**

We present a successful aortic valve replacement with cardiopulmonary bypass in a 48 years old lady with antiphospholipid syndrome, who has severe aortic regurgitation as a result of Libman-sacks endocarditis. Antiphospholipid antibodies were positive and the clinical data showed both negative cultures and infective parameters. Surgically resected vegetations revealed sterile fibrinous and verrucous vegetations on aortic valve. Valve replacement and the course of cardiopulmonary bypass was uneventful, and the patient was discharged well.

**Conclusions:**

Classically Libman-Sacks endocarditis is often and more commonly associated with autoimmune diseases such as systemic lupus erythematosus, although it can occur in both primary and secondary antiphospholipid syndrome. It is not a common entity, and it is a frequent underestimated disease as most clinicians do not routinely screen for valvular lesion in patients with antiphospholipid syndrome unless they are symptomatic. However, due to its high prevalence of cardiac involvement, clinicians should have a high index of suspicion in the attempt to minimize cardiovascular and haemodynamic complications. Valve surgery in patients with antiphospholipid syndrome carries considerable early and late morbidity and mortality, usually caused by thromboembolic and bleeding events. The perioperative anticoagulation management and haemostatic aspect of antiphospholipid syndrome present an exceptional challenges to clinicians, surgeons, anaesthetists and laboratory personnel.

## Background

Antiphospholipid syndrome (APS) is a rare coagulative disorder with antiphospholipid antibody mediated pro-thrombotic state mainly characterised by hypercoagulable complications. Cardiac manifestations of APS include arterial or venous thromboses, valve diseases, coronary artery disease, intracardiac thrombus, pulmonary hypertension and dilated cardiomyopathy, with the functional impairment of heart valves being the most common manifestation, which commonly associated with Libman-Sacks endocarditis. Libman-Sacks endocarditis, also known as non-bacterial thrombotic, verrucous, or marantic endocarditis, originally described in patients with systemic lupus erythematosus, is a well-known complication of APS. We present a successful aortic valve replacement in a 48 years old lady with aortic valve Libman-sacks endocarditis and APS who presented with acute pulmonary oedema.

## Case presentation

A 48 years old lady was admitted to cardiology ward complaining of progressively worsening breathlessness, orthopnoea and chest discomfort for 1 month. Coexisting medical conditions include chronic hypertension on treatment, with 2 episodes of spontaneous miscarriages previously and 1 episode of transient ischemic attack 3 years ago.

Clinically, she was haemodynamically stable with a wide pulse pressure. Finger clubbing was noted with livedo reticularis rashes over bilateral upper and lower limbs without ulcers or thrombophlebitis. Her peripheral pulses were bounding with water-hammered pulse, and a grade III diastolic murmur was heard during cardiac auscultation. In addition, she has elevated jugular venous pressure, crepitation over both lung bases, and bilateral pedal oedema.

**Fig. 1:**
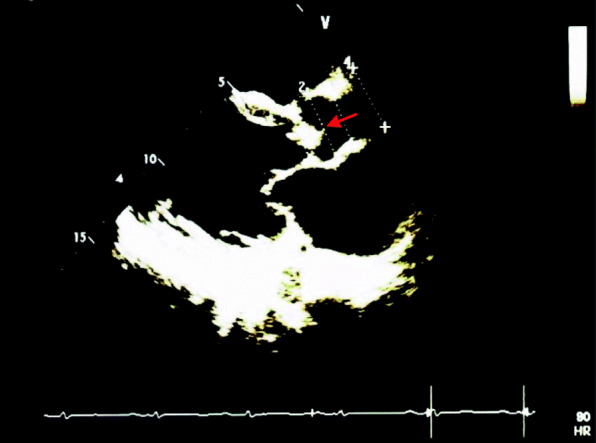
Transthoracic echocardiography shows thickened aortic leaftlets with vegetations attached to aortic cusps (red arrow)

Echocardiography showed dilated left atrium and left ventricles with preserved ejection fraction. Severe aortic regurgitation was found with PHT of 156 ms, mean pressure gradient of 32 mmHg, and end diastolic velocity of 29 cm/s. In addition, a vegetation sized 1.2cm^2^ was seen on aortic valve (Fig. 1). Mild mitral regurgitation was also noted with thickened anterior leaflet. There was minimal pericardial effusion seen but no intracardiac thrombus. Electrocardiography showed normal sinus rhythm with left ventricular hypertrophy and P-mitrale. Coronary angiography was subsequently done which revealed minor coronary disease.

Blood investigation revealed bicytopenic picture of anemia and thrombocytopenia, with elevated activated partial thromboplastin time (aPTT) and Erythrocyte sedimentation rate (ESR). Otherwise the reports were not suggestive of occult infection with normal white cell count, C-reactive protein (CRP), and negative blood cultures. D-dimer was positive but there was no features suggestive of deep venous thrombosis or pulmonary embolism. Further serological investigations revealed positive antinuclear antibody (1:640), Lupus anticoagulant (LA), anti-smith antibodies, direct and indirect coombs’ test. Subsequent bone marrow aspiration and cytogenetic study showed a grossly normal specimen. She was thus treated as acute pulmonary oedema secondary to severe aortic regurgitation, precipitating by aortic valve vegetation with newly diagnosed antiphospholipid syndrome. Therapeutic enoxaparin therapy was initiated perioperatively.

**Fig. 2:**
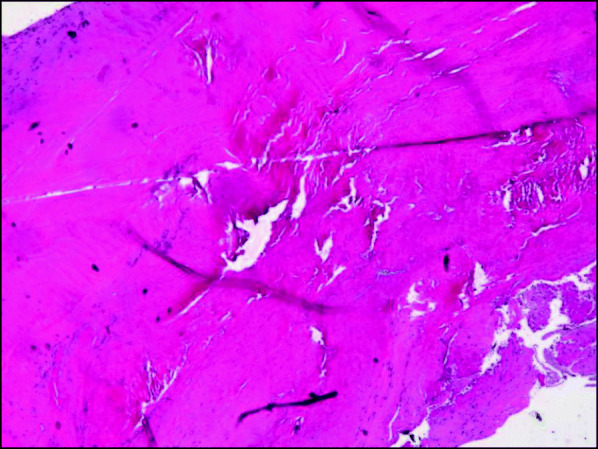
Eosinophilic amorphous material composed of fibrin and platelet thrombi attached to the valve as vegetation. There is no bacterial colonies or fungal organisms identified (Hematoxylin and Eosin, 20X)

She underwent aortic valve replacement, and intraoperative exposure of aortic valve revealed verrucous thickening of aortic leaflets with vegetations involving all three cusps, with rolled edge on left coronary cusp. No perforation or destruction of cusp tissue was identified. A mechanical valve was implanted following the excision of aortic valve. The course of cardiopulmonary bypass was uneventful. Subsequent microscopic findings of the valve specimen confirmed the diagnosis of Libman-Sacks endocarditis (Fig. 2).

Postoperatively, oral anticoagulation therapy was started with international normalized ratio (INR) target of 2.5 to 3.5 with subcutaneous enoxaparin as bridging therapy. There was no excessive bleeding or major thromboembolic complications occurred during in-patient stay. Corticosteroid therapy was started at 2 months postoperatively by Hematology team. She was well and free of complications at 6 months and 1 year follow-up. Clinical examinations and echocardiography demonstrated satisfactory aortic valve function. The microbiological culture of the excised vegetations revealed sterile specimens.

## Discussion

### Identifying patients with Antiphospholipid syndrome (APS)

Antiphospholipid syndrome (APS) is a multi-system disorder of autoimmune aetiology. This syndrome is defined clinically by arterial and venous thrombotic events as well as recurrent pregnancy loss, with serologically positive antiphospholipid antibodies including lupus anticoagulant (LA) and anticardiolipin antibodies (aCL). It was originally described and most often found in patients with systemic lupus erythematosus (SLE), however, even patients without features of autoimmune disease may harbour antiphospholipid antibodies and suffer from thromboembolic events, thus leading to the distinction of primary and secondary APS. APS affects 2% of population and SLE is found in 40% of patients with APS [1]. The prevalence of antiphospholipid antibodies increase with age, especially elderly patients with coexisting chronic diseases [2]. In fact, the prevalence of antiphospholipid antibodies in normal population was reported at 2% with the mean age of 39 years but increased to 12% in patients over 65 years of age [2]. Nevertheless, the definite diagnosis of APS requires the presence of both clinical symptoms and positive serological markers [3].

### Valvular manifestation in Antiphospholipid syndrome

APS comprises a wide array of clinical features such as venous and arterial thromboses, recurrent pregnancy loss and even thrombocytopenia. Heart valve disease is the most common cardiac manifestation in patients with APS, and it is defined in the absence of rheumatic and infective endocarditis. It was reported as high as 32–38% of valvular lesion occur in patients with APS by transthoracic echocardiography [4, 5]. When transoesophageal echocardiography is used, the incidence may increase up to 75% [6]. Thus, approximately one third of patients with APS exhibit valvular abnormalities, with significantly greater prevalence in secondary APS rather than primary APS [7]. Patients with APS often suffer from valvular heart disease which is defined by its morphological pattern of valvular thickening and vegetation, also known as Libman-sacks endocarditis [4]. Classically Libman-Sacks endocarditis is described in the context of SLE, being the most characteristic cardiac manifestation of this autoimmune disease, affecting 35–65% of patients with SLE [8]. Later on, lesions similar to those described by Libman and Sacks also found to be associated with APS, be it primary or secondary APS [5, 9]. Libman-Sacks endocarditis has a predilection for mitral valve, followed by aortic valve [4]. The predominant functional abnormalities ranging from valvular thickening to valvular regurgitation, whereas stenosis is rarely seen. In this case, the negative infective parameters and cultures with the finding of sterile fibrinous and verrucous vegetations confirmed the diagnosis of Libman-Sacks endocarditis.

The role of antiphospholipid antibodies in the pathogenesis of Libman-Sacks endocarditis remained unclear, probably the result of autoimmune antibodies being directed against the negatively charged phospholipids on the endothelial membranes, either due to micro injuries secondary to stress or turbulence, or induction of autoantibodies by molecular mimicry caused by infectious agents [9, 10]. Microscopy of Libman-Sacks endocarditis revealed deposition of immunoglobulins and complement components, suggesting a possible interaction with surface antigens leading to subendocardial inflammation and subsequent thrombosis and fibrosis [11]. It has also been proposed that these antibodies merely promote thrombus formation on the injured valve endocardium rather than a more direct pathogenic role, leading to the formation of fibrin-rich thrombi on the injured valve [12]. ,Grossly, Libman-Sacks valvular lesions are typically small sessile and wart-like growth which form a fibrous plaque with focal calcification. This process is accompanied by marked scarring and fibrosis, which organize and coalesce resulting in the thickening and distortion of the valve, and subsequent valvular dysfunction, findings which are evident in this case study [8, 13].

Patients with APS often present with thromboembolic events from other systems rather than cardiac manifestation, most commonly cerebrovascular ischemic events, which is apparent in this case study where patient had transient ischemic attack years ago. These thromboembolic episodes might be due to direct effect of antiphospholipid antibodies or intermittent dislodgement of Libman-Sacks vegetations. Majority of valvular impairment associated with Libman-Sacks endocarditis are mild with minor insignificant haemodynamic disturbance without clinically overt disease [9, 14]. Only 4–6% of APS patients with heart valve disease develop severe valvular dysfunction, mostly severe regurgitation, that requires surgical intervention [15].

Diagnosing Libman-Sacks endocarditis often necessitates the exclusion of rheumatic valve disease and infective endocarditis. Most clinicians do not routinely screen for valvular lesion in APS patients unless the patient is symptomatic or in the presence of new murmur. Recently, the international consensus committee has revised the definition of APS associated cardiac valve lesion with the following criteria: Coexistence of laboratory criteria of APS along with echocardiography detection of lesions, and/or regurgitation and/or stenosis of mitral or aortic valve, with the defining valve lesions of thickness more than 3 mm, localized thickening involving the leaflet’s proximal or middle portion, and irregular nodules on the atrial face of the edge of the mitral valve, and/or the vascular face of the aortic valve [16]. However, it must be remembered that this valve lesion may predisposes to infective endocarditis. In our case, both the aortic and mitral valves leaflets were thickened. Interestingly, the mitral valve was relatively spared with only mild regurgitation likely due to eccentric hypertrophy of left ventricle caused by chronic aortic regurgitation and the resultant volume overload.

Unlike infective endocarditis where the valve needs to be completely excised to remove infected tissue, repair and preservation of the valve is possible in selected patients with Libman-Sacks endocarditis, thus eliminating the need for lifelong anticoagulation therapy. The indications for surgery remain dispute, and the outcomes of surgical valvular replacement have been limited to case reports or series. However, we agreed on the clear indications for surgical intervention, which include severe valvular dysfunction, large vegetations and recurrent embolization despite therapeutic anticoagulation. Furthermore, non-bacterial thrombotic endocarditis may have much greater surgical risk for vegetation embolization than infective endocarditis due to the risk of thromboembolism [17]. To date, there are still conflicting data regarding the role and effect of antiplatelet and corticosteroid on regression of valvular lesions in APS, but these therapies may prevent embolic events [18]. In our patient, corticosteroid therapy was not initiated in immediate postoperative period as intraoperative cardiopulmonary bypass course was uneventful with stable postoperative recovery course, but it may be warranted in long term as an empirical treatment of APS, in our case, at 2nd month postoperatively to prevent further thromboembolic events.

Aortic valve replacement is deemed necessary due to severe valvular regurgitation causing significant symptomatic ventricular dysfunction. The selection of mechanical valve in this case was based on the existing unavoidable need for lifelong anticoagulation therapy. A mechanical valve may be theoretically more advantageous over a tissue valve considering the younger age of patients at the time of surgery in regards to the risk of structural valve deterioration, and the need for lifelong anticoagulation. However, the use of tissue valve has increased progressively over the years to allow for easier and safer monitoring and management of anticoagulation therapy, with regards to prevention of thromboembolic events and bleeding complications, compounded by the complex monitoring of anticoagulation due to presence of antibodies, and the possible coexisting thrombocytopenia [19]. This is particularly significant in patients with higher risk of coagulopathy and thromboembolic events. Further studies need to be done to study the outcomes of both types of valve, and if tissue valve is used, the possible immunological deterioration of the valve.

### Perioperative Management of Haemostasis and Anticoagulation Therapy

It is well recognized that patients with APS undergoing surgical valve replacement surgery with cardiopulmonary bypass are at much greater risk of thrombotic and bleeding episodes. Perioperative management of patients with APS undergoing cardiac surgery is a major concern and remains challenging due to significant risk of thrombosis with the cessation of anticoagulation therapy; as well as bleeding secondary to excessive anticoagulation or coagulation factor deficiency [20]. Furthermore, the stress associated with surgery, systemic inflammatory response syndrome (SIRS) and sepsis, or a minor alteration in anticoagulation regime can trigger catastrophic APS, which in term can cause thrombotic occlusion in multiple organs resulting in mortality as high as 50% [1, 21]. Deep hypothermic cardiac arrest further predispose APS patients to these complications. Our patient is fortunate as surgery was successful with uneventful perioperative period. Enoxaparin was used instead of unfractionated heparin to avoid the associated complication of heparin-induced-thrombocytopaenia, and therapeutic dose of enoxaparin was started preoperatively prior to surgery, and continued postoperatively as bridging therapy to reduce the risk of thromboembolic event while minimizing haemorrhagic complication.

## Conclusion

Libman-Sacks endocarditis is not a common entity and is frequently underestimated as majority valvular involvement in patients with APS are asymptomatic and often diagnose incidentally. It is difficult to detect Libman-Sacks endocarditis, and the diagnosis often requires strong clinical suspicion with exclusion of more common valve lesions such as rheumatic or infective endocarditis. We hope that this case study is able to emphasize Libman-Sacks endocarditis as a cause of valvular heart disease not only in systemic lupus erythematosus, but also in primary APS. Early detection and prompt treatment of valvular disease in APS may halt the progression of valvular dysfunction since valve replacement surgery in APS carries significant perioperative morbidity and mortality.

## Data Availability

Not applicable.

## Abbreviations

aCL: Anti-Cardiolipin Antibodies
ACT: Activated Clotting Time
APS: Antiphospholipid Syndrome
aPTT: Activated Partial Thromboplastin Time
CRP: C-Reactive Protein
ENA: Extractable Nuclear Antigen
ERO: Effective Regurgitant Orifice
ESR: Erythrocyte Sedimentation Rate
INR: International Normalized Ratio
LA: Lupus Anticoagulant
PHT: Pressure Half-Time
SIRS: Systemic Inflammatory Response Syndrome
SLE: Systemic Lupus Erythematosus
